# Use of Interactive Food Labels to Increase Confidence in Preparing Produce among College Students

**DOI:** 10.3390/nu16152507

**Published:** 2024-08-01

**Authors:** Kim Spaccarotella, Sasmita Mishra, Liam Healy

**Affiliations:** Department of Biological Sciences, Kean University, 1000 Morris Ave., Union, NJ 07083, USA; samishra@kean.edu (S.M.); healyliam95@gmail.com (L.H.)

**Keywords:** produce, young adults, self-efficacy, cooking videos, food labels, QR codes

## Abstract

College students may have limited access to produce and may lack confidence in preparing it, but cooking videos can show how to make healthy dishes. The Cognitive Theory of Multimedia Learning suggests that learning is enhanced when visual and auditory information is presented considering cognitive load (e.g., highlighting important concepts, eliminating extraneous information, and keeping the video brief and conversational). The purpose of this project was to pilot test a food label for produce grown at an urban university and assess whether student confidence in preparing produce improved after using the label and QR code to view a recipe video developed using principles from the Cognitive Theory of Multimedia Learning. The video showed a student preparing a salad with ingredients available on campus. Students indicated the label was helpful and reported greater perceived confidence in preparing lettuce after viewing the label and video (mean confidence of 5.60 ± 1.40 before vs. 6.14 ± 0.89 after, *p* = 0.016, *n* = 28). Keeping the video short and providing ingredients and amounts onscreen as text were cited as helpful. Thus, a brief cooking video and interactive label may improve confidence in preparing produce available on campus. Future work should determine whether the label impacts produce consumption and if it varies depending on the type of produce used.

## 1. Introduction

Although consuming produce provides many health benefits [1], the intake of produce among college students varies. According to the 2022 American College Health Association survey, about 27.8% of students reported eating an average of three or more servings of vegetables per day [2], and students may consume a greater percentage of white potatoes and starchy vegetables and a lower percentage of dark green, orange or other vegetables [3]. A variety of factors may influence produce consumption and healthy eating in this population, including cost, lack of facilities and tools for cooking and preparing fruits, vegetables, and other healthy ingredients, and lack of access to a traditional grocery store and taste [4]. In addition, food agency, the ability to plan and successfully prepare foods in varied environments and circumstances may impact dietary intake [5]. Food agency includes factors such as lack of confidence or self-efficacy in obtaining and preparing food, and greater cooking confidence has been associated with higher consumption of fruits and vegetables and lower consumption of fried potatoes in adults [5].

Several strategies have been suggested to remove barriers, improve food preparation skills, and help college students eat more produce and healthy foods. Increased availability of produce on campus, for example, through campus food pantries or farmstands, may improve access and reduce the costs of healthy eating for students [5]. Although these resources are a relatively new concept on campuses, and there is limited research on them, in general, food pantry clients have expressed interest in increased access to produce and simple recipes to help them prepare healthy foods [6], and students report that having access to free produce through campus farmstands helps them to consistently consume a nutrient-dense diet [7]. Additional resources have included classes [8], recipe cards or booklets, and cooking videos [9,10]. In particular, videos have the advantage of providing on-demand, cost-effective learning and may enhance participants’ learning and successful completion of a cooking task when paired with a recipe [11]. Feedback from students has shown they prefer videos that are approachable, short, use inexpensive ingredients, provide detailed captioning, and are delivered through a platform such as Facebook [11,12]. Participants have reported that videos improve their comprehension of and comfort with the cooking process, help them acquire new skills, and add to their enjoyment of food preparation [13]. They also liked being able to pause the video and replay sections as needed [8,13], and this may help decrease the likelihood of information overload when a large amount of oral and visual information is presented together. In addition, the Cognitive Theory of Multimedia Learning recommends that highlighting important material on screen using text or symbols, removing information that does not contribute to learning or reinforce the concepts being taught, and keeping the lesson brief with a conversational tone further enhance learning [14].

Research using these resources with college students is limited but has shown promising results. For example, a study of 29 college food pantry clients provided students with weekly, cost-effective produce recipes and ingredients over 6 weeks and found that female participants significantly increased their produce consumption [9]. Among college sophomores (*n* = 65), participation in four 2-h cooking classes and a supermarket tour significantly improved attitudes toward cooking compared to students who attended a 1-h cooking demonstration [15]. In Brazil, six weeks of cooking classes and a supermarket tour with freshmen university students (*n* = 38) significantly improved self-efficacy in preparing produce compared to students in the control group, who continued their usual routine [16]. Research has also explored the use of currently available materials, such as Supplemental Nutrition Assistance Program-Education (SNAP-Ed) resources, with college students and highlighted the need to tailor materials to support college students’ unique needs, including strategies for cooking with limited time and supplies, incorporating culturally appropriate foods and language that is relevant to college students [17]. Similarly, a review of 28 studies with adults that included cooking assignments, classes, or demonstrations reported positive effects on dietary intake, health outcomes (e.g., serum cholesterol), and nutrition knowledge [18]. Finally, the use of cooking videos to demonstrate preparation of lasagna supported participants (*n* = 141) as they learned new cooking skills and increased their enjoyment of meal preparation compared to those who received only a recipe card [13] and were also perceived as helpful from young adults (*n* = 34) learning to cook calcium-rich foods [11].

Interactive food labels are another novel tool that may be useful in nutrition education to guide participants to specific resources and information related to food production and nutrition [19]. For example, Quick Response (QR) codes can be easily added to a food package to provide this information and guide users to further details about recipes, preparation techniques, and cooking videos made with particular ingredients. Research has found that consumers perceive interactive food labels as valuable [20] but may not be motivated to scan the QR code on their own without additional prompts [21]. In addition, a high perceived fit between the QR code and the product may increase the likelihood that consumers will scan the code [22]. However, research on QR code use to increase produce intake among students is limited. Thus, the purpose of this project was to assess whether access to produce and a label with nutrition information, recipes, and other interactive features (e.g., QR code to a brief video demonstrating how to prepare a healthy produce recipe) helps students improve confidence in preparing produce and to gain feedback on the features they would find most helpful in the video and interactive label.

## 2. Materials and Methods

To explore the feasibility of making local produce accessible to students, leaf lettuce was grown in the controlled conditions of the campus greenhouse. According to the International Produce Association, lettuce is in the top five most common vegetables grown in the United States [23]. Because leaf lettuce seeds germinate quickly compared to other salad crops, it is easy to maintain continual lettuce harvests [24], and growing lettuce inside a greenhouse allows year-round harvests when it is too hot or cold to grow lettuce outdoors. In addition, leaf lettuce can be easily prepared by students with limited cooking skills using pantry ingredients and basic kitchen tools and equipment; thus, it was chosen for use in the current study. To ensure consistent nutrient content, each batch of lettuce was cultivated under the same growth conditions, starting from transplanting seedlings to harvest. Plants were irrigated with tap water, and no additional fertilizer was used. Previous research has found that food pantry clients often use Google for recipe ideas when they want to learn how to prepare less familiar produce [25]. Thus, to identify recipes to prepare using the greens, a Google search was completed using the phrase “lettuce recipes”, and the first five results were selected. These were reviewed to identify one (a blueberry peach salad) that seemed easy to prepare, appealing to students, utilized pantry staples (e.g., canned peaches in 100% fruit juice, oil, vinegar, and honey) in addition to lettuce, could be easily made in a dormitory with minimal cooking equipment and could be modified to meet current Dietary Guidelines [1] (e.g., reducing salt and honey in the recipe to lower sodium and sugar content, respectively). To ensure students could relate to the cooking demonstration, a student researcher worked with the principal investigators to create a script and develop a recipe video. To increase the effectiveness of the video, the Cognitive Theory of Multimedia Learning was used to guide its design. The video was brief (4 min and 30 s) [26] so that learners could focus on a small amount of information at a time and minimize extraneous material (e.g., elaborate backgrounds or music) [14]. Textboxes were used onscreen to highlight important information, such as an ingredient list, and each step was both shown and narrated to enhance learning [14]. To engage students, the narrator also used enthusiastic, conversational language with the goal of building a partnership with the viewers [14].

**Figure 1:**
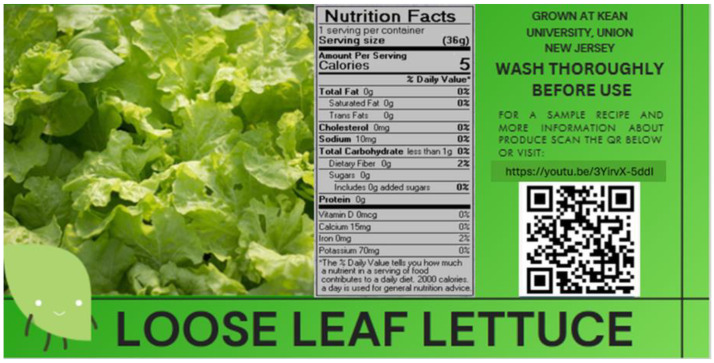
Interactive food label sample for lettuce.

A QR code for the video was then created and added to the food label to provide users with further information about the product. Then, users were invited to give feedback on the label and video. A nutrition facts panel for looseleaf lettuce was generated using FoodWorks 18 (The Nutrition Company, Long Valley, NJ, USA) and was also added to the label (Figure 1).

The label was placed on the containers used to package the lettuce as part of salad kits for the campus food pantry with ingredients needed to make the recipe shown in the video. An image of the label was also sent via email blast to the campus community with an announcement letting students know that fresh produce was being grown on campus and inviting them to give feedback on the label if they wished. There were no additional requirements for participation in the survey. This method was chosen to reduce bias by ensuring students from a variety of majors and backgrounds represented on campus had the opportunity to participate. Moreover, the student body is diverse, with 65% identifying as Black/African American, Native American/Alaskan Native, Asian, or Hispanic/Latino in 2023 [27]; thus, the data collected would be relevant to other mid-sized, urban research campuses with a diverse student body. Interested students could scan the QR code or visit the URL given on the label to view the video and answer a brief, anonymous electronic survey. The survey included sections asking about usual fruit and vegetable intake, adapted from the Short Healthy Eating Index Survey [28], self-efficacy in preparing produce, adapted from the Cooking and Food Provisioning Action Scale [29], and the students’ thoughts on the label and video. The research was deemed exempt by the Kean University Institutional Review Board, and all participants gave written informed consent. A power analysis was conducted using G*Power 3.1 [30], and it was determined that a sample size of 26 would give 80% power (*p* < 0.05) to detect differences in cooking self-efficacy before and after viewing the interactive label and cooking video. To account for missing data and skipped questions, additional students were recruited. All other data analysis was conducted using IBM SPSS Statistics 29 (IBM, Armonk, NY, USA), and all values are reported as means ± standard deviations unless otherwise noted.

## 3. Results

Thirty-five students provided data on confidence in preparing produce, and twenty-eight gave feedback about the label and video. About 40 containers of lettuce and salad kits were shared with the pantry between October 2023 and April 2024. Of the students surveyed, one indicated that they had used the campus food pantry. Though most participants appeared to view the label and video after receiving an email blast about the study rather than scanning a container of lettuce at the food pantry, verbal feedback from food pantry staff towards the label was very positive, and they indicated that the students they served were excited to receive the lettuce and salad kits.

On average, students consumed less than two servings per day of fruit and vegetables (Table 1). Questions about self-efficacy in preparing produce were answered using a 7-point Likert scale with options of 1, “strongly disagree”, to 7, “strongly agree” [22]. The mean confidence in dealing with unexpected results was approximately neutral (4.74 ± 1.50). Participants somewhat agreed that it is easy to accomplish the desired result (5.34 ± 1.41) and solve problems that arose when preparing produce (5.63 ± 1.14).

Before viewing the interactive label, the mean confidence in preparing the salad greens shown on the label in the container was 5.60 ± 1.40, and participants somewhat agreed that they were comfortable preparing the produce. Because the data on mean confidence in preparing lettuce before and after watching the video were not normally distributed, the Wilcoxon Signed Rank Test was used to test for significant differences [31], and bootstrap confidence intervals were calculated [32]. After viewing the video, the mean confidence in preparing the lettuce was significantly greater (6.14 ± 0.89, *p* = 0.016, effect size = 0.32). They rated the label’s ability to help students select and prepare produce they receive at the campus food pantry a 3.83 ± 0.93 (on a scale of 1, “not at all helpful”, to 5, “extremely helpful”) (95% CI 3.47–4.18). Participants indicated that the label was “informative”, “intuitive and easy to understand”, and “straightforward and to the point”. Three students shared concerns: the label might not be widely used by students, students do not typically read labels, and the QR code should be advertised more “excitingly”.

When participants were asked for qualitative feedback on the video, suggestions included adding more close-ups of ingredient preparation, chopping lettuce into small pieces, filming the video in a kitchen instead of at a dining room table, showing text with ingredient amounts on-screen and speeding up parts of the video that showed longer steps such as chopping and mixing. One student commented that they liked the use of canned peaches since “canned fruit is often thought of as less healthy”. Another liked being able to navigate to different sections of the video depending on which step of the recipe they were working on. They felt the video “[gave] people more ideas of how to prepare food, especially if they don’t normally cook” and was “helpful”. They also indicated that the videos could encourage students to try a new dish: “I felt as though I’d be more comfortable preparing my own dish with a recipe video. Recipe videos can also give inspiration for other meals that I can make with similar ingredients”.

## 4. Discussion

Based on the feedback collected, the interactive label and video were generally perceived by students as helpful, and brief videos with captions highlighting keywords were preferred, as suggested by the Cognitive Theory of Multimedia Learning [11]. Principles of the Cognitive Theory of Multimedia Learning were applied to the development of the video in several ways [14]. The theory suggests that learning is most effective when both visual and auditory information is provided without overwhelming the student, maximizing the brain’s ability to temporarily store and process the information in the working memory [14]. Thus, the video included both a visual cooking demonstration and narration. It was also designed to reduce cognitive effort, such as confusing, distracting, or unnecessary information, by highlighting key ingredients onscreen and using a simple background without music. Finally, to help viewers manage the intrinsic load or the inherent difficulty of the subject matter, the video was designed to be brief, and viewers could pause and replay sections to control the amount of new information they received at one time.

Similar to previous findings, participants suggested that narration should not last for the entire length of the video and provided positive feedback that could be used to refine it [11].

Prior research has reported that about 35–36% of college students and young adults use a nutrition label always or often [33,34], although among adults 20 years and older, 4 of 5 reported using the Nutrition Facts panel when choosing food to purchase [35], suggesting that additional use may occur for students some of the time. Thus, although food labels provide useful nutrition information, many students may not use them consistently. However, label use may be associated with improved diet quality and eating behaviors. Among middle and high school students who always or almost always used the Nutrition Facts panel of the food label when choosing foods, there were significantly greater odds of consuming healthy foods compared to those who reported sometimes or never using the Nutrition Facts panel [36]. Further, college students who more frequently used nutrition labels were significantly more likely to consume more fruits and vegetables compared to those who read labels sometimes or rarely [35]. In addition, nutrition label use and attitude toward preparing healthy meals significantly predicted healthy eating behaviors [34], suggesting a link between nutrition label use and well-being. The Dietary Guidelines recommend consuming 2.5 cups of vegetables and 2 cups of fruit daily for a 2000-calorie diet [1], and students in the current study consumed below these recommendations. Additional research is needed to understand whether the use of an interactive food label increases produce consumption so that it is closer to recommendations. In the current study, feedback from participants suggested some students may perceive canned goods as less healthy. Interventions that feature healthy pantry staples, such as canned goods with less sugar, sodium, and fat, in recipes with produce may help overcome this stigma and expand students’ options for consuming fruits and vegetables to meet dietary recommendations [37].

Research on cooking demonstrations for college students and their impact on self-efficacy is limited. However, college sophomores participating in cooking classes significantly increased their confidence in using various cooking techniques [15]. Furthermore, previous research with adults over 16 years of age found that an 8-week cooking class significantly improved cooking confidence [38], as well. Similarly, the current study found student confidence in preparing produce significantly increased after viewing the cooking video and label. Future research should clarify whether these findings translate into increases in produce consumption, as well.

Research on QR code use suggests that visually complex QR codes and advertising may overwhelm some consumers but intrigue those who are more curious and that a good perceived fit of the QR code to the product also influences intent to scan [18]. Thus, although many participants liked the QR code, others felt it was not “exciting” or would not be used by their peers, indicating that apathy may be a key issue for some users. Similarly, previous findings from research with QR code follow-through found catering to less curious consumers may be best for increasing scanning [22] by balancing a presented QR code with just enough information to clearly state what benefits scanning would provide. Additional feedback from students could also help clarify which types of QR code designs are most successful for increasing interest in produce in this population.

Previous research has also indicated that providing food pantry users with healthy cooking kits that include recipe ingredients as a bundle may improve the selection of healthy foods and that attractive packaging and labels indicating that the ingredients are “healthy” may improve client interest in trying these foods [6]. Further, having an opportunity to prepare the recipes as opposed to watching a demonstration only may increase the likelihood that long-term behavior change will occur [11]. In the present study, students generally believed the video and label were helpful. However, participants were students at a diverse, medium-sized, urban university [39], which limits generalizability to other types of institutions and student populations. In addition, the students who provided feedback may have volunteered due to greater interest in food labels, produce, or cooking compared to those who did not respond. Given the smaller size of the campus food pantry to which the lettuce was provided in the current study (the pantry served about 15 students per week at the time of the study), further research should test the interactive label at a larger campus pantry and determine whether the use of recipe videos and cooking kits impacts produce consumption among college students using campus food pantries, farm stands or other resources seeking to make produce accessible to students. Pilot testing at additional schools would also provide feedback from a wider range of students with different demographics, backgrounds, and needs and, potentially, a broader range of interest levels in healthy cooking and interactive food labels.

## 5. Conclusions

Our findings suggest that an interactive food label with a QR code to a recipe video is perceived as helpful by university students and may increase confidence in preparing produce such as salad greens. Using a brief format that includes ingredient information on-screen and highlights key steps may assist students in preparing produce that they receive as part of campus programs to increase the accessibility of healthy ingredients. In the present study, participants consumed less produce than the recommendations; future research should assess the effects of an interactive label on produce intake among university students and those who use the campus food pantry to better target interventions towards these groups. In addition, research should determine students’ preferences for types of recipes and produce so the intervention can be tailored to the needs of students, which may vary across campuses and regions or based on the size of the institution. The long-term effects of interactive label use on produce consumption and confidence in preparation should also be studied. Future research should clarify if these effects and preferences vary based on other characteristics, such as year of study, major, or gender. Given the many health benefits produce provides and the flexibility of a recorded cooking demonstration for users who may not be able to come to an in-person class, the interactive food label may help campuses bring produce to more students while increasing their confidence in preparing it.

## Figures and Tables

**Table 1:** Mean produce consumption and preparation skills.

Variable ^1^	Mean ± SD	95% CI
Average fruit servings per day (*n* = 34)	1.76 ± 1.35	1.29–2.24
Average vegetable servings per day (*n* = 34)	1.91 ± 1.31	1.45–2.37
Confident in dealing with the unexpected	4.74 ± 1.50	4.23–5.26
Believe that it is easy to accomplish desired results	5.34 ± 1.41	4.86–5.83
Able to solve problems when preparing produce	5.63 ± 1.14	5.24–6.02
Comfortable with produce preparation in general	5.60 ± 1.40	5.12–6.08
Comfortable preparing loose leaf lettuce before viewing interactive label (*n* = 28)	5.46 ± 1.43	4.93–5.93
Comfortable preparing loose leaf lettuce after viewing interactive label (*n* = 28) *	6.14 ± 0.89	5.79–6.43

^1^ Response options for the self-efficacy questions ranged from 1 (strongly disagree) to 7 (strongly agree); *n* = 35 except where noted. SD—standard deviation; CI—confidence interval. * *p* = 0.016; effect size = 0.32.

## Data Availability

The datasets presented in this article are not readily available due to privacy concerns and institutional data protection guidelines.
